# Prognostic Value of Fibrinogen among COVID-19 Patients Admitted to an Emergency Department: An Italian Cohort Study

**DOI:** 10.3390/jcm9124134

**Published:** 2020-12-21

**Authors:** Pierpaolo Di Micco, Vincenzo Russo, Novella Carannante, Michele Imparato, Giuseppe Cardillo, Corrado Lodigiani

**Affiliations:** 1 UOC Medicina, Fatebenefratelli Hospital of Naples, 80131 Naples, Italy; micheleimparato@gmail.com; 2 Department of Translational Medical Sciences, University of Campania “Luigi Vanvitelli”—Monaldi Hospital, Piazzale Ettore Ruggeri, 80131 Naples, Italy; v.p.russo@libero.it; 3 Emergenza Infettivologica—Pronto Soccorso Ospedale Cotugno, AO dei Colli, 80131 Naples, Italy; carannantenovella@gmail.com; 4 Medylab, Biochimica Avanzata Laboratory, 80131 Napoli, Italy; giuseppe.cardillo.75@gmail.com; 5 Thrombosis and Hemorrhagic Center, Humanitas Clinical and Research Center, IRCCS, 20089 Rozzano, Italy; corrado.lodigiani@humanitas.it

**Keywords:** COVID-19, SARS-CoV-2, hemostasis, disseminated intravascular coagulation, fibrinogen, D-dimer

## Abstract

Introduction: A highly pathogenic human coronavirus able to induce severe acute respiratory syndrome (SARS) has been recently recognized as the cause of the coronavirus disease 2019 (COVID-19); the disease became pandemic after a few months. Little is still known about the laboratory prognostic markers in COVID-19 patients. The aim of our study was to describe the prognostic value of clotting parameters for the prediction of severe form of COVID-19 characterized by acute respiratory distress syndrome (ARDS) at hospital admission. Material and Methods: From a large cohort of 152 patients consecutively admitted from February to March 2020 for fever and dyspnea to the emergency departments (ED) of three Italian hospitals, we evaluated 85 patients with confirmed diagnosis of COVID-19 and 67 patients with acute illness. All patients underwent medical history checks, physical examination, and laboratory evaluation. Prothrombin time (PT), activated thromboplastin time (aPTT), fibrinogen and D-dimer tests were performed and compared, first, between COVID-19 and control groups, and then between COVID-19 patients with or without ARDS. Results: COVID-19 patients were more likely to show abnormal baseline levels of PT, aPTT, D-dimer, and fibrinogen at admission compared to the control group. COVID-19 patients with ARDS showed a statistically significant increase in levels of fibrinogen compared to those without ARDS (720 (621–833) vs. 490 (397.5–601.5); *p*= 1.8653 × 10^−9^ (0.0765). A cut-off value of 617 mg/dL had a sensitivity of 76% and a specificity of 79% in identifying COVID-19 patients with ARDS. Conclusion: A serum level of fibrinogen of 617 mg/dL in COVID-19 patients admitted to emergency department may help to identify early those with ARDS.

## 1. Introduction

The coronavirus disease 2019 (COVID-19) outbreak became pandemic because of the easy transmission of severe acute respiratory syndrome human coronavirus-2 (SARS-CoV-2) [1]. COVID-19 is associated with relevant morbidity and mortality, in particular if complicated by SARS, rapidly evolving toward acute respiratory distress syndrome (ARDS) or venous thromboembolism (VTE) [2,3,4,5]. Italy is among the countries majorly hit by COVID-19, with more than 229,000 laboratory-confirmed cases [6]. From early worldwide reports to the present, SARS-CoV-2 infection has been associated with early laboratory alterations [7,8]; moreover, clotting abnormalities have been associated with COVID-19 complications and prognosis [9,10,11,12,13,14]. There is a growing interest in the role of biomarkers in the screening and early detection of SARS-CoV-2 infection at emergency departments. The aim of our study was to describe the prognostic value of clotting parameters for the prediction of severe form of COVID-19 characterized by ARDS at hospital admission.

## 2. Materials and Methods

From a large cohort of 152 patients consecutively admitted from February to March 2020 for fever and dyspnea to the emergency departments (ED) of three Italian hospitals (Humanitas Clinical and Research Center of Milan, Cotugno Hospital of Naples, Fatebenefratelli Hospital of Naples), we selected 85 patients with confirmed diagnosis of COVID-19; 67 consecutive patients with acute illness admitted to ED were used as a control group. All patients underwent medical history checks, physical examination, and laboratory evaluation. Laboratory tests included prothrombin time (PT) expressed as seconds and international normalized ratio (INR), activated thromboplastin time (aPTT), and fibrinogen and D-dimer tests. The selection methods were similar to those used in our previous reports [13].

Clotting tests were performed according to standard methods and commercial kits [14]; in particular, the values of D-dimer and fibrinogen were measured in human citrated plasma on IL CoagulationSystem^®^ (Instrumentation Laboratory, Bedford, MA, USA). D-dimer was measured bymeans of an immunoturbidimetric latex-particle assay, and fibrinogen by means of the functional clotting assay according to Clauss. Fibrinogen and D-dimer levels higher than 400 mg/dL and 500 μg/dL, respectively, were considered pathological.

The detection of SARS-CoV-2 by real-time quantitative reverse-transcription polymerase chain reaction (RT-PCR) assay on nose/throat swab or sputum samples has been used to achieve the laboratory confirmation of COVID-19 [15]. ARDS diagnosis was defined according to the Berlin definition [16]. The COVID-19 population was divided into two groups according to the diagnosis of isolated pneumonia (No ARDS Group) or pneumonia with ARDS (ARDS Group). The clotting factor values were also compared between the two groups in order to describe their prognostic value in identifying COVID-19 patients with ARDS at ED admission. The institutional ethics committee of the Fatebenefratelli Hospital of Naples approved the protocol (FBGID-90320). Verbal and written informed consent for participation was provided for all patients.

### Statistical Analysis

The Anderson–Darling test was used to analyze data normality. Continuous variables were reported using median and interquartile intervals (IQR). Categorical variables were indicated as frequency counts and percentages. Differences between groups were evaluated using the 2-tailed Fligner–Policello and Fligner–Killeen test for continuous data, and Barnard’s or Fisher’s test for categorical variables. A receiver-operating characteristics (ROC) curve was constructed to identify the parameter and its cut-off points that can best predict ARDS diagnosis at admission. In all statistical tests, the alpha value was set to 5%. Statistical comparisons were performed using the statistical software package MatLab R2018a (The MathWorks Inc., Natick, MA, USA).

## 3. Results

**Figure 1:**
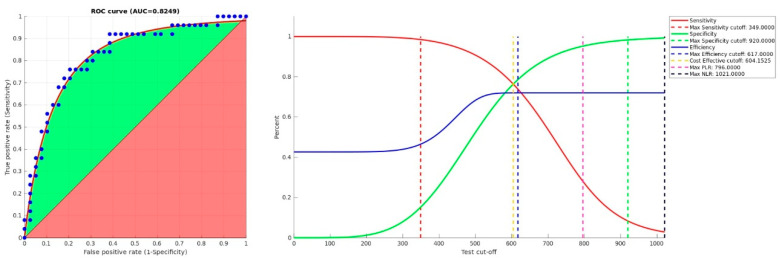
ROC curves of fibrinogen as a predictor of COVID-19 ARDS. On the left, the green area is the gain above the chance, represented by a red area of 0.5; on the right, sensitivity (red full line), specificity (green full line), and efficiency (blue full line) are functions of fibrinogen concentration (mg/dL). Dashed lines show cut-offs where parameters are maximized: at 349 mg/dL, sensitivity is max (in red); at 604 mg/dL, sensitivity and specificity are equal (in yellow); at 617 mg/dL, efficiency is max (in blue); at 796 mg/dL, positive likelihood ratio is max (in magenta); at 920 mg/dL, specificity is max (in green); at 1021 mg/dL, negative likelihood ratio is max (in black).ROC: receiver-operating characteristics; AUC: area under curve; PLR: Positive Likelihood Ratio; NLR: Negative Likelihood Ratio.

There were no statistically significant differences in demographic characteristics between the COVID-19 and control group (Table 1); however, COVID-19 patients showed a statistically significant increase in values of PT, aPTT, D-dimer, and fibrinogen compared to controls (Table 2). In the COVID-19 group, patients with ARDS (*n*: 34) did not show differences in demographic characteristics, sex distribution, PT, aPTT, or D-dimer values compared to those without ARDS (*n*: 54) (Table 3); however, an increased fibrinogen median value has been shown in COVID-19 patients with ARDS (Table 4). ROC curve analysis revealed that fibrinogen was an independent predictor of ARDS at admission (area under curve (AUC) = 0.83, 95% CI: 0.71–0.93, *p =* 5.2421 × 10^−9^) (Table 5); a cut-off value of 617 mg/dL had a sensitivity of 76% and a specificity of 79% (Figure 1).

During hospitalization with a median follow-up of 26 (IQR: 11–43) days, eight deep venous thrombosis (DVT) and three pulmonary embolism (PE) events occurred among the study population. Eight patients died, among them two following PE. The numbers and rates of DVT, PE, death following PE, and overall death among the groups are summarized in Table 6.

## 4. Discussion

From early reports to the present, alterations of hemostasis with a trend toward hypercoagulable state have been reported in COVID-19 patients [7,8]; in particular, increased levels of D-dimer and fibrinogen have been associated with poor prognosis among hospitalized patients [9,10,11,12,13], mainly due to pulmonary embolism or disseminated intravascular coagulation (DIC) [17]. However, the blood samples were usually collected during hospitalization, and little is still known about the role of clotting parameters in identifying, at ED admission patients, with more severe form of COVID-19, characterized by ARDS, and their relationship with thromboembolic events [18]. Our study confirms significant differences in clotting parameter levels between COVID-19 patients and control group at ED admission (13) and supports the hypothesis that the hypercoagulable state is present since the early stage of the disease and may lead to venous thrombosis complications and disseminated intravascular coagulation (DIC) [19]. Among COVID-19 patients, fibrinogen levels higher than 617 mg/dL are more likely associated with those presenting with severe clinical form of the disease characterized by ARDS at admission and at increased risk of venous thromboembolism events during hospitalization, in particular pulmonary embolism and PE-related death. Our findings suggest a possible role for fibrinogen-level testing to identify early the clinically severe form of COVID-19 at admission to the emergency department; in particular, a cut-off of 617 mg/dL might be used to early identify those with ARDS, with a sensitivity of 76% and a specificity of 79%. According to these findings, routine testing of clotting factors should be integrated with current triage strategies for patients with suspected COVID-19 [20] in order to collect more detailed information for their clinical management. The potential association between antithrombotic therapy and the clinical presentation of COVID-19 is still debated [21], and further studies are necessary to evaluate the possible role of clotting factors, in particular fibrinogen, in guiding the use of antiviral and antithrombotic therapies [22,23,24] as part of routine medical therapy during hospitalization.

## 5. Conclusions

A serum level of fibrinogen of 617 mg/dL in COVID-19 patients admitted to an emergency department may help to identify early, with a sensitivity of 76% and a specificity of 79%, those with ARDS.

## Figures and Tables

**Table 1:** Demographic characteristics of the study population.

Patients’ Characteristics	Controls *n*: 67	COVID-19 *n*: 85	*p*-Value
Males, *n* (%)	47 (70%)	58 (68%)	0.83715
Age <40 years, *n* (%)	2 (3%)	4 (5%)	0.77552
Age 40–60 years, *n* (%)	19 (28%)	26 (31%)	
Age >60 years, *n* (%)	46 (68%)	55 (65%)	

For sex, we used a 2-tailed Barnard’s exact test; for age, a 2-tailed Fisher’s exact test.

**Table 2:** Comparisons of laboratory parameters between Control and COVID-19 groups.

Parameters	Controls *n*: 67	COVID-19 *n*: 85	FP Test *p*-Value	FK Test *p*-Value
PT INR, median (IQR)	1.11(1.01–1.16)	1.13(1.07–1.21)	0.0198	0.1149
aPTT ratio, median (IQR)	0.96(0.88–1.02)	0.99(0.90–1.11)	0.0290	0.0159
D-dimerμg/dL, median (IQR)	505(276–650)	637(412–964)	0.0112	0.1049
Fibrinogen mg/dL, median (IQR)	450(356–591)	589(461–721)	0.0001	0.1922

All tests are 1-tailed. FP: Fligner–Policello; FK: Fligner–Killeen. PT: Prothrombin time. INR: international normalized ratio; aPTT: Activated partial thromboplastin time.

**Table 3:** Demographic characteristics of the study population according to the presence or not of acute respiratory distress syndrome (ARDS).

Patients’ Characteristics	Overall *n*: 85	No ARDS Group *n*: 54	ARDS Group *n*: 31	*p*-Value
Males, *n* (%)	58 (68%)	37 (68%)	21 (68%)	1.0000
Age <40 years, *n* (%)	4 (5%)	3 (6%)	1 (3%)	0.9645
Age 40–60 years, *n* (%)	26 (31%)	16 (31%)	10 (32%)	
Age >60 years, *n* (%)	55 (65%)	35 (65%)	20 (65%)	

For sex, we used a 2-tailed Barnard’s exact test; for age, a 2-tailed Fisher’s exact test.

**Table 4:** Comparisons of laboratory parameters between COVID-19 patients without and with acute respiratory distress syndrome (ARDS).

Parameters	Overall *n*: 85	No ARDS Group *n*: 54	ARDS Group *n*: 31	FP Test *p*-Value	FK Test *p*-Value
PT INR, median (IQR)	1.13 (1.07–1.21)	1.13 (1.07–1.19)	1.14 (1.09–1.25)	0.1312	0.6040
aPTT ratio, median (IQR)	0.99 (0.90–1.11)	0.975 (0.900–1.095)	0.99 (0.89–1.115)	0.3128	0.4395
D-dimer μg/dL, median (IQR)	637 (412–964)	569.5 (341.75–954.75)	698 (508.75–1255)	0.0670	0.5034
Fibrinogen mg/dL, median (IQR)	589 (461–721)	490 (397.5–601.5)	720 (621–833)	1.8653 × 10^−9^	0.0765

All tests are 1-tailed. FP: Fligner–Policello; FK: Fligner–Killeen. PT: Prothrombin time. INR: international normalized ratio; aPTT: Activated partial thromboplastin time.

**Table 5:** Area under the fibrinogen receiver-operating characteristics curve estimation.

AUC	Standard Error	Confidence Interval		Comment	Standard AUC	*p* Value
0.82488	0.05677	0.71361	0.93615	Good test	5.7227	5.2421 × 10^−9^
Maximized Parameter				Cut-off (mg/dL)		
Sensitivity				349		
Cost-Effective				604		
Efficiency				617		
Positive Likelihood Ratio				796		
Specificity				920		
Negative Likelihood Ratio				1021		

ROC: receiver-operating characteristics; AUC: area under curve.

**Table 6:** Numbers and rates of clinical outcome events in the study population.

Clinical Outcome Events	Overall *n*: 85	No ARDS Group *n*: 54	ARDS Group *n*: 31	*p*-Value
DVT, *n* (%)	8 (9.4)	4 (7.4)	4 (12.9)	0.41
PE, *n* (%)	3 (3.5)	0 (0)	3 (9.7)	0.02
Death for PE, *n* (%)	2 (2.4)	0 (0)	2 (6.4)	0.06
Overall death, *n* (%)	6 (7.1)	1 (1.8)	5 (16.1)	0.01

DVT: deep venous thrombosis; PE: pulmonary embolism.
